# Classical Macrophage Activation Up-Regulates Several Matrix Metalloproteinases through Mitogen Activated Protein Kinases and Nuclear Factor-κB

**DOI:** 10.1371/journal.pone.0042507

**Published:** 2012-08-03

**Authors:** Wei-Chun Huang, Graciela B. Sala-Newby, Angela Susana, Jason L. Johnson, Andrew C. Newby

**Affiliations:** 1 Bristol Heart Institute, University of Bristol, Bristol, United Kingdom; 2 Cardiovascular Center, Kaohsiung Veteran General Hospital, Kaohsiung City, Taiwan; 3 Taiwan School of Medicine, National Yang Ming University, Taipei City, Taiwan; Medical University Innsbruck, Austria

## Abstract

Remodelling of the extracellular matrix (ECM) and cell surface by matrix metalloproteinases (MMPs) is an important function of monocytes and macrophages. Recent work has emphasised the diverse roles of classically and alternatively activated macrophages but the consequent regulation of MMPs and their inhibitors has not been studied comprehensively. Classical activation of macrophages derived *in vitro* from un-fractionated CD16^+/−^ or negatively-selected CD16^−^ macrophages up-regulated MMP-1, -3, -7, -10, -12, -14 and -25 and decreased TIMP-3 steady-state mRNA levels. Bacterial lipopolysaccharide, IL-1 and TNFα were more effective than interferonγ except for the effects on MMP-25, and TIMP-3. By contrast, alternative activation decreased MMP-2, -8 and -19 but increased MMP -11, -12, -25 and TIMP-3 steady-state mRNA levels. Up-regulation of MMPs during classical activation depended on mitogen activated protein kinases, phosphoinositide-3-kinase and inhibitor of κB kinase-2. Effects of interferonγ depended on janus kinase-2. Where investigated, similar effects were seen on protein concentrations and collagenase activity. Moreover, activity of MMP-1 and -10 co-localised with markers of classical activation in human atherosclerotic plaques *in vivo*. In conclusion, classical macrophage activation selectively up-regulates several MMPs *in vitro* and *in vivo* and down-regulates TIMP-3, whereas alternative activation up-regulates a distinct group of MMPs and TIMP-3. The signalling pathways defined here suggest targets for selective modulation of MMP activity.

## Introduction

The matrix metalloproteinases (MMPs) are a group of structurally-related enzymes that have a catalytic Zn^2+^ ion and are subject to inhibition by complexing with tissue inhibitors of metalloproteinases (TIMPs) [1]. The enzymes have overlapping specificities for a large spectrum of ECM components. A few MMPs (including MMPs-1, -8, -13, -14 and -19) can cleave fibrillar collagens, whereas others cleave denatured collagens, proteoglycan core proteins and elastin [1]. Several MMPs that attach to cell surface proteins and the so-called membrane-type MMPs (MMP-14 to -17, -25, and -26) that are intrinsic membrane proteins, mediate pericellular proteolysis. MMPs may also cleave cell surface and soluble proteins or release factors sequestered in the ECM [1]. Finally, several of the MMPs have the ability to cleave and activate the pro-forms of other MMPs [1].

Through their effects of the ECM, MMPs promote the egress of leukocytes from bone marrow and their invasion into foci of inflammation [2]. Moreover, cleavage of matrix and non-matrix proteins, including several mediators of inflammation [3], affects proliferation, migration and death of leucocytes [2], [4]. For this reason there is great interest in the regulation of MMP production in monocytes and macrophages. Much recent work has focussed on the diversity of macrophage behaviour. At one extreme, macrophages may be *classically activated* by Toll-like receptor ligands and pro-inflammatory mediators, including tumour necrosis factor-α (TNFα), interleukin-1 (IL-1) and interferonγ (IFNγ); at the other they may be *alternatively activated* by distinct mediators, including IL-4 and IL-13 [5], [6]. During inflammation, for example, classically activated macrophages effectively clear infectious organisms and also orchestrate angiogenesis and the ingress of connective tissue cells to form a granuloma, events that could depend on ECM remodelling by MMPs [2]. During subsequent healing, alternatively activated macrophages may encourage connective tissue cells to reform the ECM [5], [6], which also requires tightly-regulated proteolysis [2]. In chronic inflammatory states including persistent infections, auto-immune diseases and situations of repeated physical or biological injury remodelling of the ECM by MMPs can be more extensive and irreversible [7]. In extreme cases, the ECM may lose its structural integrity leading to mechanical failure. Examples include periodontal disease [8], arthritides [9] and the complications of tuberculosis [10]. In advanced atherosclerosis, MMPs can contribute to plaque rupture and myocardial infarction [11], which is the leading cause of death in advanced societies.

Defining the spectrum and mechanisms of MMP production from macrophages might help develop therapies for all these pathologies. Two previous studies surveyed the MMP and TIMP system in monocytes [12], [13] but their pattern of expression in macrophages and the effects of classical and alternative activation have not been previously reported. We therefore conducted a comprehensive *in vitro* study on the regulation of MMPs and TIMPs in macrophages and the signalling pathways involved and then validated some major conclusions in human atherosclerotic plaques *in vivo*.

## Materials and Methods

Reagents included Ficoll-Paque Plus (GE healthcare, Little Chalfont, Bucks, UK) RPMI, FBS, DPBS (Invitrogen, Paisley, UK) MACS monocyte isolation kit II (Miltenyi Biotec, Bisley, Surrey, UK). QuantiTect Reverse Transcription Kit and Quanti Tect SYBR Green PCR Kit (Qiagen, Crawley, W. Sussex, UK). SC514 was obtained from Cayman Chemicals (Cambridge Bioscience, Cambridge, UK), whereas LY 294002, PD98059, SB20380, SP600125 and JAK inhibitor IV, [3-amino-5-(N-tert-butylysulfonamido-4-phenyl)-indazole] (ABSPI), were obtained from Merck Chemicals (Beeston, Notts, UK); all were dissolved in DMSO. Recombinant human interferonγ (IFNγ), interleukin-4 (IL-4) and macrophage colony stimulating factor (MCSF) were from R&D systems (Abingdon, Oxon, UK). Fatty acid free bovine serum albumin (BSA) for cell treatments was obtained from Roche (Welwyn Garden City, Herts, UK). LPS (*E.coli* 026:B6) and all other reagents and primers were purchased from Sigma-Aldrich (Gillingham, Dorset, UK). The following antibodies were used: MAPKs, AKT (S_473_), NF-κBp65(S_536P_), STAT-6 (Y_641P_) and STAT1(total and Y_701P_), were from New England Biolabs (Herts, UK), MMP-14 (AB8221), TIMP-3 (MAB3318) and GAPDH (MAB374) (Millipore, Watford, UK), MMP-10 (MAB9101)and CD206 (AF2534) from R&D, MMP-12 from Abcam (ab38935, Cambridge, UK), COX-2 (SC-19999) and IκBα (SC-371) from Santa Cruz (Heidleberg, Germany) and HRP-labelled secondary antibodies from Sigma-Aldrich.

Monocytes were isolated from buffy coats from healthy blood donors, which were collected from National Blood Transfusion Service (Bristol, UK) or from heparinised blood of healthy volunteers after written informed consent under National Research Ethics Service approval from Frenchay Research Ethics Committee reference 09/H0107/22 and South West 4 Research Ethics Committee reference 10/HO102/72, respectively. Unselected CD16^+/−^ monocytes cells were isolated using Ficoll-Paque Plus, cleared of erythrocytes and allowed to adhere to plastic for 2 hours. CD16^−^ monocytes were purified by negative selection using MACS monocyte isolation kit II according to the manufacturer's instructions. Monocyte maturation was performed in RPMI 1640 containing 10% FCS and 20 ng/mL of MCSF for 7 days and the medium was replaced on day 4. To polarize macrophages, complete RPMI media with 5% foetal bovine serum was supplemented with recombinant human IFNγ (20 ng/mL) and LPS (100 ng/mL) or interleukin-4 (20 ng/mL) [14]. Conditioned medium and cell extracts for RNA and protein were then obtained and subjected to real time quantitative PCR western blotting or zymography as described previously [13]. Primer sets are recorded in Table 1. MMP-1 total and active was captured by antibody binding and quantified using the Fluorokine™E kit from R&D, according to the manufacturer's instructions

**Table 1:** Primers for quantitative RT-PCR.

Primer		Sequence	Annealing temp (°C)	Fragment size (bp)
18S	Forward	CTCGATGCTCTTAGCTGAGT	56	300
	Reverse	CTTCAAACCTCCGACTTTCG		
MMP-1	Forward	TGTCACACCTCTGACATTCACCAA	58	162
	Reverse	AAATGAGCATCCCCTCCAATACCT		
MMP-2	Forward	CCATTGAACAAGAAGGGGAACTTG	60	182
	Reverse	GGATACCCCTTTGACGGTAAGGAC		
MMP-3	Forward	CGCCTGTCTCAAGATGATATAAAT	60	152
	Reverse	CTGACAGCATCAAAGGACAA		
MMP-7	Forward	GCTTTAAACATGTGGGGCAAAGAG	60	286
	Reverse	CAGAGGAATGTCCCATACCCAAAG		
MMP-8	Forward	AAAAGCATATCAGGTGCCTTTCCA	60	194
	Reverse	CAGCCACATTTGATTTTGCTTCAG		
MMP-9	Forward	GAGGCGCTCATGTACCC	58	300
	Reverse	CGATGGCGTCGAAGATG		
MMP-10	Forward	CAAAATCTGTTCCTTCGGGATCTG	58	299
	Reverse	GATGCCTCTTGGATAACCTGCTTG		
MMP-11	Forward	GGGCTGAGTGCCCGCAACCGAC	60	236
	Reverse	TCCCCATGCCAGTACCTGGCGAAGT		
MMP-12	Forward	TTACCCCCTTGAAATTCAGCAAGA	60	225
	Reverse	CGTGAACAGCAGTGAGGAACAAGT		
MMP-14	Forward	GGAGACAAGCATTGGGTGTT	58	343
	Reverse	GGTAGCCCGGTTCTACCTTC		
MMP-17	Forward	ACGAGGCCTGGACCTTCCGCTCCT	60	216
	Reverse	ACTCCCGCACACCGTACAGCTGCC		
MMP-19	Forward	CCGAGTCTACTTCTTCAAGGGCAA	60	180
	Reverse	GTGGTATCCAAGGTTATGCCCGTA		
MMP-23	Forward	GGGACCACTTCAACCTCACCTACA	60	175
	Reverse	GGGTAGAAGCCTATCCGGAGGTC		
MMP-25	Forward	GGGCGTGGACTGGCTGACTCGCTA	60	166
	Reverse	ACGCATGGTGGCCACTGTCCCTGG		
TIMP-1	Forward	GATACTTCCACAGGTCCCACAACC	60	160
	Reverse	CAGCCAACAGTGTAGGTCTTGGTG		
TIMP-2	Forward	GAAGGAAGTGGACTCTGGAAACGA	60	234
	Reverse	ATGAAGTCACAGAGGGTGATGTGC		
TIMP-3	Forward	CTTCCGAGAGTCTCTGTGGCCTTA	60	230
	Reverse	CTCGTTCTTGGAAGTCACAAAGCA		
36B4	Forward	GCCAGCGAAGCCACGCTGCTGAAC	60	76
	Reverse	CGAACACCTGCTGGATGACCAGCCC		
COX-2	Forward	GCCATGGGGTGGACTTAAATCATA	60	168
	Reverse	CAGGGACTTGAGGAGGGTAGATCA		
CD206	Forward	CGGTGACCTCACAAGTATCCACAC	58	216
	Reverse	TTCATCACCACACAATCCTCCTGT		
MAF-1	Forward	GAGGGACGCGTACAAGGAGAAATA	60	266
	Reverse	TAGGTGGTTCTCCATGACTGCAAA		
SOCS-3	Forward	CCCCCAGAAGAGCCTATTACATCT	60	149
	Reverse	GTACTGGTCCAGGAACTCCCGAAT		
IKK2	Forward	TGAGGATGAGAAGACTGTTGTCCG	60	295
	Reverse	CGTGAAACTCTGGTCTTGTTCCCT		
IκB (Hu)	Forward	CTACTGGACGACCGCCACGACAGC	60	91
	Reverse	CGAGGCGGATCTCCTGCAGCTCCTTG		
IκB (Pig)	Forward	CTGGTGTCGCTCTTGTTGAAGTGT	60	208
	Reverse	GCAGCTCATCCTCTGTGAACTCTG		

Human coronary artery specimens were collected from cadaveric heart donors to the Bristol Coronary Artery Biobank under National Research Ethics Service approval from Frenchay Research Ethics Committee reference 08/H0107/48. The left and right coronary arteries were dissected within 48 hours of death and pressure fixed at 100 mmHg with 4% paraformaldehyde for 24 hours at 4°C. After paraffin embedding, serial 3 or 5 µm sections were used for immunocytochemistry using the methods described previously [15] and the following antibodies; CD68, Dako M0876; MMP-1, Millipore MAB3307; MMP10, R& D MAB9101; COX-2, Abcam AB15191 and NF-κB, Abcam, ab31481. For dual staining antibodies were labelled to yield either a red fluorescent product at the site of the antigen (Alexifluor 594TM) or a green fluorescent product at the site of the antigen (Alexifluor 488TM). DAPI was then used in order to fluorescently label nuclei blue. The specificity of the immunostaining was demonstrated by inclusion of a negative control using isotype-specific non-immune serum or IgG.

Means were compared by ANOVA followed by Student's t-test with Bonferroni correction for multiple experimental conditions. Normally distributed data is reported as mean±SD or SEM, as indicated. Non-normally distributed data was log transformed and, if normalised, tested as above. In this case values are reported as log weighted means with 95% confidence limits in parenthesis.

## Results

### Expression of mRNAs for MMPs and TIMPs in un-stimulated, classically and alternatively activated macrophages

**Figure 1:**
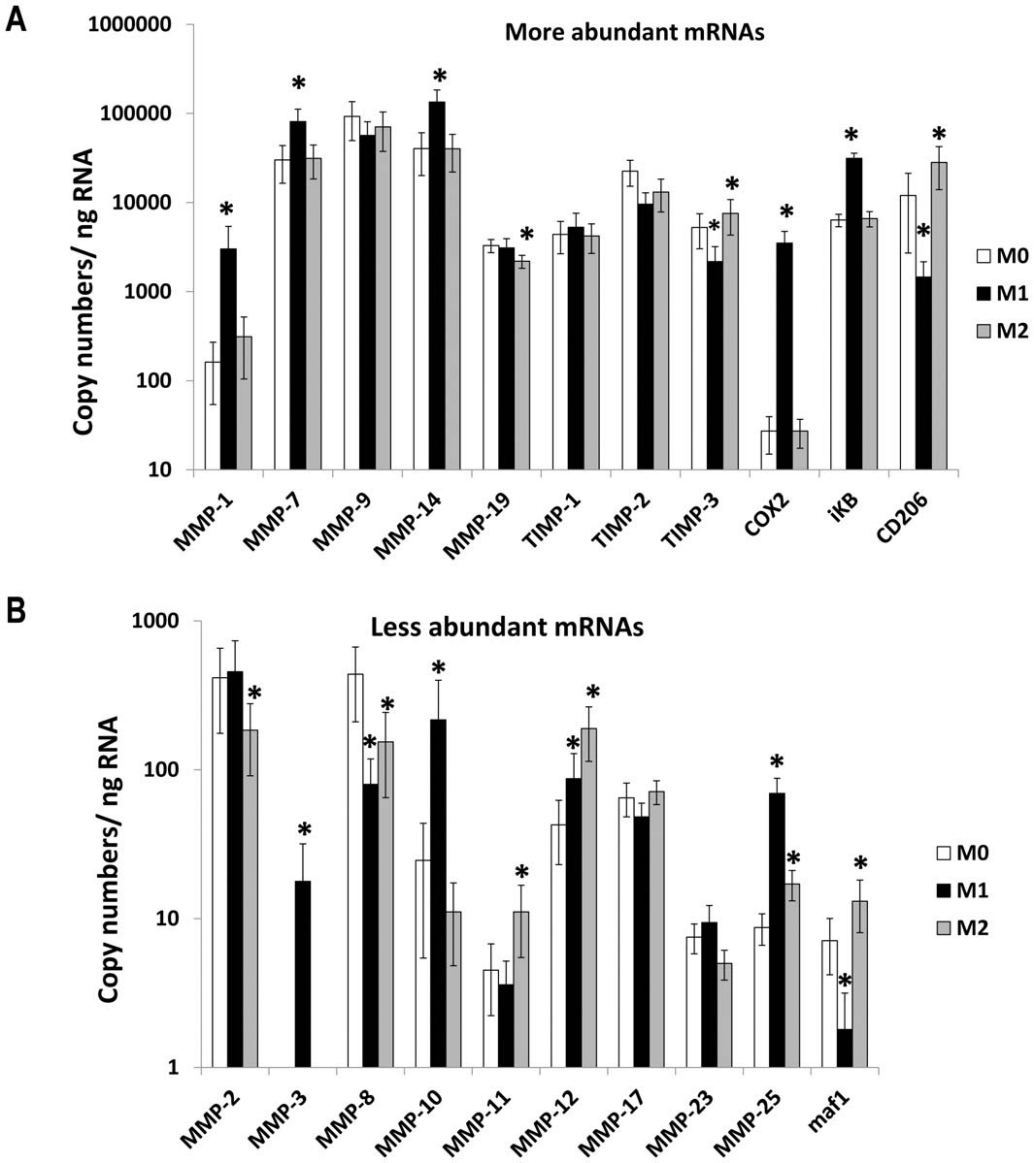
Effects of classical and alternative macrophage activation on MMP and TIMP mRNA levels. Steady state mRNA levels were measured by qPCR in M-CSF differentiated macrophages derived from unselected human CD16^+/−^ monocytes (M0) or classically activated with LPS and IFNγ (M1) or alternatively activated with IL-4 (M2) for 18 hours. Panels A and B show more and less abundant mRNAs, respectively. Values are means ± SEM * p<0.05 vs M0 (n = 7).

Based on copy numbers of mRNA, MMPs -7, -9, -14, -19 and TIMP-1, -2 and -3 were constitutively expressed at high levels in un-stimulated human macrophages differentiated with M-CSF from un-fractionated populations of CD16^+/−^ monocytes (Fig. 1A). The mRNAs for other MMPs studied were expressed at lower levels (Fig. 1B) and MMP-13 mRNA was undetectable.

Macrophages in a pro-inflammatory milieu are likely to be classically activated, and to simulate this we initially used LPS and IFNγ to produce so-called M1 macrophages (Fig. 1A, B). By contrast, during resolution of inflammation macrophages may be alternatively activated, which we simulated with IL-4 to produce M2 macrophages (Fig. 1A, B). Based on previous studies [14], cyclo-oxygenase-2 (COX-2) and IκBα were included as positive controls for classical activation and the mannose receptor (CD206) and maf1 for alternative activation. Consistent with these expectations we found that classical-activation of macrophages (M1) up-regulated COX-2 and IκBα but down-regulated CD206 and maf1 mRNAs (Fig. 1A, B). By contrast, alternative activation of macrophages (M2) up-regulated CD206 and maf1 mRNAs (Fig. 1A, B). Classical activation (M1) also up-regulated mRNAs for MMP-1, -3, -7, -10, -12, -14 and -25 and down-regulated MMP-8 and TIMP-3 mRNAs (Fig. 1A, B). Note the scales are logarithmic and that fold changes are given in Table 2. Alternative activation with IL-4 (M2 conditions) increased mRNAs for MMPs-11, -12 and -25 and TIMP-3 and down-regulated MMPs-2, -8 and -19 (Fig. 1A, B and Table 2). As a result MMP-1, -2, -3, -7, -10, -14 and -25 were increased significantly in classically versus alternatively activated macrophages (Table 2). Conversely, MMPs-11 and -12 and TIMP-3 were increased significantly in alternatively versus classically activated macrophages, whereas other MMPs and TIMPs were similar in M1 and M2 macrophages (Table 2).

**Table 2:** Comparison of quantitative RT-PCR results among macrophage phenotypes from adhered mononuclear cells and CD16- monocytes (n = 7).

	M1/M2 ratio				M1/Control ratio				M2/Control ratio			
Gene	mean	lower 95%CI	upper 95% CI	p value	mean	lower 95%CI	upper 95% CI	p value	mean	lower 95%CI	upper 95% CI	p value
MMP-1	5.7	2.1	14.9	**0.016**	9.5	4.5	20.2	**0.002**	1.7	1.2	2.4	0.055
	*4.9*	*2.7*	*8.9*	***0.002***	*7.4*	*1.8*	*31.3*	***0.032***	*1.5*	*0.6*	*3.8*	*0.397*
MMP-2	4.2	1.8	9.6	**0.014**	1.3	0.9	1.7	0.197	0.3	0.2	0.5	**0.007**
	*5.1*	*1.3*	*19.3*	***0.047***	*1.4*	*0.6*	*3.0*	*0.434*	*0.3*	*0.1*	*1.4*	*0.168*
MMP-3	324.6	42.7	2467.0	**0.003**	232.6	49.8	1086.1	**0.001**	0.7	0.4	1.3	0.363
	*1875.2*	*808.2*	*4351.4*	***<0.001***	*1632.5*	*744.1*	*3581.6*	***<0.001***	*0.9*	*0.7*	*1.1*	*0.374*
MMP-7	3.2	2.3	4.3	**<0.001**	3.6	2.4	5.4	**0.001**	1.1	0.9	1.5	0.325
	*3.5*	*2.1*	*6.1*	***<0.001***	*3.4*	*1.8*	*6.7*	***<0.001***	*0.9*	*0.7*	*1.3*	*0.792*
MMP-8	0.6	0.4	0.8	**0.017**	0.2	0.1	0.3	**<0.001**	0.3	0.2	0.5	**0.005**
	*0.3*	*0.03*	*2.3*	*0.577*	*0.3*	*0.1*	*0.6*	***0.011***	*0.3*	*0.2*	*0.6*	***0.020***
MMP-9	0.6	0.2	2.1	0.453	0.6	0.3	1.1	0.147	0.9	0.5	1.7	0.834
	*3.9*	*0.6*	*24.5*	*0.186*	*2.6*	*0.2*	*36.8*	*0.506*	*0.7*	*0.2*	*2.2*	*0.509*
MMP-10	24.6	3.9	157.0	**0.009**	12.2	2.4	60.5	**0.016**	0.5	0.1	2.3	0.400
	*15.7*	*6.0*	*40.9*	***0.001***	*9.2*	*1.7*	*48.4*	***0.037***	*0.6*	*0.2*	*2.2*	*0.444*
MMP-11	0.3	0.2	0.5	**0.006**	0.8	0.5	1.4	0.540	2.4	1.8	3.2	**0.002**
	*0.3*	*0.2*	*0.4*	***0.001***	*1.1*	*0.5*	*2.4*	*0.840*	*3.8*	*2.0*	*7.3*	***0.006***
MMP-12	0.5	0.3	0.7	**0.002**	2.5	0.8	7.4	0.142	5.1	1.7	15.4	**0.020**
	*0.3*	*0.1*	*1.0*	*0.090*	*3.6*	*1.4*	*9.5*	***0.038***	*11.3*	*4.3*	*29.4*	***0.002***
MMP-14	10.7	2.2	52.7	**0.025**	8.0	1.6	40.1	**0.041**	0.8	0.4	1.3	0.328
	*5.6*	*2.3*	*13.9*	***0.008***	*9.3*	*1.1*	*77.2*	*0.079*	*1.7*	*0.2*	*16.6*	*0.680*
MMP-17	0.7	0.5	1	0.084	0.8	0.5	1.2	0.374	1.2	0.9	1.5	0.245
	*1.3*	*0.4*	*4.2*	*0.653*	*0.6*	*0.4*	*1.1*	*0.173*	*0.5*	*0.1*	*2.4*	*0.426*
MMP-19	1.2	0.8	1.8	0.382	0.8	0.6	1.1	0.216	0.7	0.6	0.8	**0.001**
	*3.1*	*0.7*	*13.6*	*0.171*	*0.9*	*0.8*	*1.0*	*0.056*	*0.3*	*0.1*	*1.3*	*0.146*
MMP-23	1.4	0.4	5.2	0.646	0.8	0.4	1.9	0.694	0.6	0.3	1.3	0.227
	*2.8*	*0.4*	*22.5*	*0.366*	*1.2*	*0.8*	*1.7*	*0.417*	*0.4*	*0.1*	*3.0*	*0.419*
MMP-25	3.2	1.5	6.8	**0.020**	7.1	3.6	14	**0.001**	2.2	1.6	3.1	**0.004**
	*2.6*	*1.4*	*5.1*	***0.015***	*10.9*	*6.5*	*18.6*	***<0.001***	*4.2*	*2.0*	*8.7*	***0.005***
TIMP-1	0.8	0.4	1.7	0.549	0.9	0.6	1.3	0.530	1.1	0.7	1.6	0.641
	*1.0*	*0.7*	*1.5*	*0.888*	*0.9*	*0.7*	*1.1*	*0.216*	*0.8*	*0.5*	*1.5*	*0.553*
TIMP-2	1.2	0.3	3.9	0.811	0.5	0.3	0.7	**0.008**	0.4	0.1	1.7	0.269
	*1.0*	*0.5*	*2.2*	*0.918*	*0.9*	*0.4*	*2.0*	*0.813*	*0.9*	*0.8*	*1.0*	*0.074*
TIMP-3	0.3	0.1	0.5	**0.004**	0.4	0.3	0.7	**0.007**	1.6	1.2	2.2	**0.014**
	0.2	0.1	0.5	**0.023**	0.5	0.1	2.1	0.395	3.4	1.4	8.6	**0.035**
maf1	0.1	0.03	0.2	**0.001**	0.1	0.1	0.3	**0.001**	1.7	1.2	2.5	**0.033**
	*0.1*	*0.03*	*0.1*	***<0.001***	*0.2*	*0.1*	*0.5*	***0.013***	*3.5*	*2.2*	*5.6*	***0.002***
CD206	0.05	0.03	0.1	**<0.001**	0.2	0.1	0.6	**0.015**	5.3	3.0	9.1	**0.001**
	*0.2*	*0.1*	*0.5*	***0.008***	*0.4*	*0.2*	*0.9*	*0.064*	*2.2*	*0.6*	*8.1*	*0.289*
COX2	140.6	85.4	231.2	**<0.001**	114.0	67.6	192.2	**<0.001**	0.8	0.6	1.1	0.183
	*177.3*	*124.8*	*251.7*	***<0.001***	*137.2*	*62.9*	*299.3*	***<0.001***	*0.8*	*0.4*	*1.5*	*0.482*
IκB	3.9	2.3	6.7	**<0.001**	3.9	2.4	6.6	**<0.001**	1	0.8	1.2	0.928
	*4.4*	*1.9*	*10.3*	***0.001***	*3.2*	*1.7*	*6.3*	***0.001***	*0.7*	*0.4*	*1.3*	*0.306*

Values for negatively-selected CD16- monocytes are shown in italics in the shaded cells. Significant p values are in bold type.

A major population of CD16^−^ monocytes and several minor CD16^+^ populations occur in human blood [16]. We observed similar levels of MMPs and TIMPs in un-stimulated macrophages derived from CD16^+/−^ populations of adhered mononuclear cells or negatively selected CD16^−^ monocytes (Table 2). Furthermore, similar effects of classical and alternative activation were seen with macrophages differentiated from mixed CD16^+/−^ or CD16^−^ monocytes (Table 2). Occasionally significant differences in one or other of CD16^+/−^ and CD16^−^ monocytes were merely trends in the other preparation (Table 2). Hence, none of these MMPs or TIMP mRNAs appeared to be exclusively expressed or up-regulated in macrophages derived from CD16^+^ monocytes.

### Effects of LPS and IFNγ separately and comparison with other classical activators, IL-1 and TNFα

**Figure 2:**
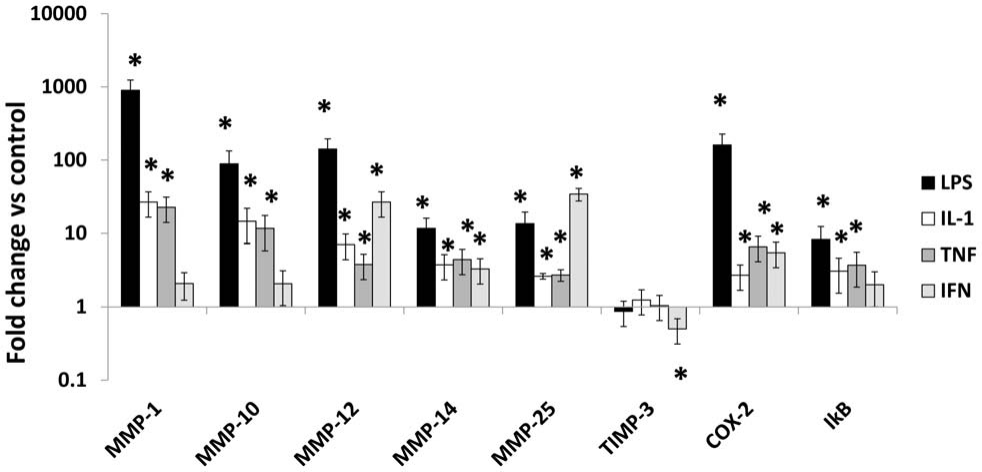
Effects of classical activators on MMP and TIMP mRNA levels. Steady state mRNA levels were measured by qPCR in M-CSF differentiated macrophages derived from unselected human CD16^+/−^ monocytes. Cells were kept un-stimulated or treated with LPS (100 ng/mL), IL-1α (20 ng/mL) TNFα (10 ng/mL), or IFNγ (100 ng/mL) for 18 hours. Steady state mRNA levels were measured and the ratio between treated cells and M-CSF alone was calculated. Values are means ± SEM * p<0.05, n = 7.

LPS on its own was a strong activator of COX-2, IκBα and MMPs-1, -10, -12, -14 and -25 but did not affect TIMP-3 (Fig. 2). LPS also up-regulated MMP-3 from undetectable starting values (results not shown). Maximal concentrations of IL-1 and TNFα were weaker activators than LPS (Fig. 2). IFNγ increased COX-2 and MMPs-12 and -14 mRNAs significantly, albeit to a lesser extent than other classical activators. IFNγ was more effective than the other classical activators tested in increasing MMP-25 and decreasing TIMP-3 mRNAs (Fig. 2).

### Role of MAP kinases, and NF-κB in MMP and TIMP up-regulation

**Figure 3:**
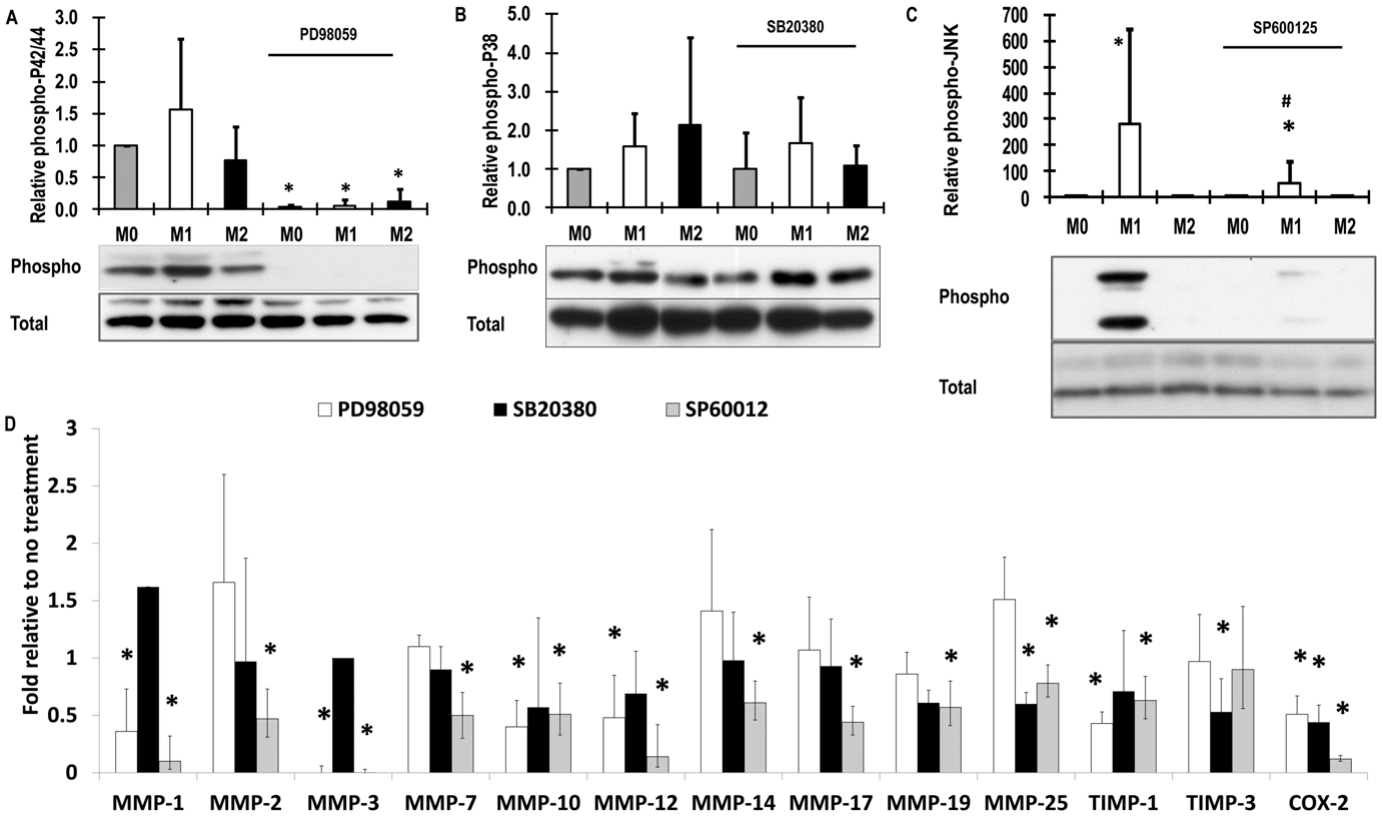
Activation of MAP kinases and effects of inhibitors. M-CSF differentiated macrophages derived from unselected human CD16^+/−^ monocytes (M0) were pre-treated for 45 min with 10 µM PD98059, SB20380, SP600125 or vehicle (0.1% v/v DMSO) and then some were classically activated with LPS and IFNγ (M1). Phospho and total proteins were measured by western blotting. Levels were normalised to total proteins levels. (A) ERK1/2 with and without PD98059, (B) p38 MAPK with and without SB20380, (C) JNK with and without SP600125, (D) Steady-state mRNA levels of MMPs and other genes were measured after 18 hours of stimulation with LPS and IFNγ. Values are log weighted means and 95% confidence intervals * p<0.05 compared to without inhibitor (n = 7).

We reported previously that constitutive expression of MMPs and TIMPs from M-CSF differentiated macrophages (M0 conditions) was not affected by inhibitors of ERK, p38 or JNK MAP kinases or IKK2 [13]. Nevertheless, production of many inflammatory mediators from macrophages depends on activation of MAP kinases leading to activation of the activator protein-1 (AP-1) transcription factor and of inhibitor of κB kinase-2 (IKK2) leading to activation of NF-κB. We therefore used well characterised, selective inhibitors to investigate whether the same is true for MMPs up-regulated in classically-activated macrophages. M-CSF differentiated macrophages had constitutive levels of phospho-ERK1/2 and p38 MAP kinase that were not further increased by classical or alternative activation (Fig. 3A, B). However, phospho-JNK was stimulated under M1 conditions (Fig. 3C). Selective inhibition of MEK with PD98059 and JNK with SP600125 inhibited ERK1/2 and JNK phosphorylation, respectively (Fig. 3A, C). SB20380 did not inhibit the p38 phosphorylation (Fig. 3B), as expected, since it is a direct inhibitor of p38 kinase activity. After induction by LPS and IFNγ (M1 conditions) MEK inhibitor PD98059 decreased mRNA levels of positive control gene COX-2 and MMPs-1, -3, -10, and -12 and TIMP-1 (Fig. 3D). JNK inhibitor SP600125 reduced mRNA levels of all these proteins plus MMPs-2, -14, -17, -19 and -25 (Fig. 3D). By contrast, p38 inhibitor SB20380 only reduced levels of COX-2, MMP-25 and TIMP-3 mRNA (Fig. 3D).

**Figure 4:**
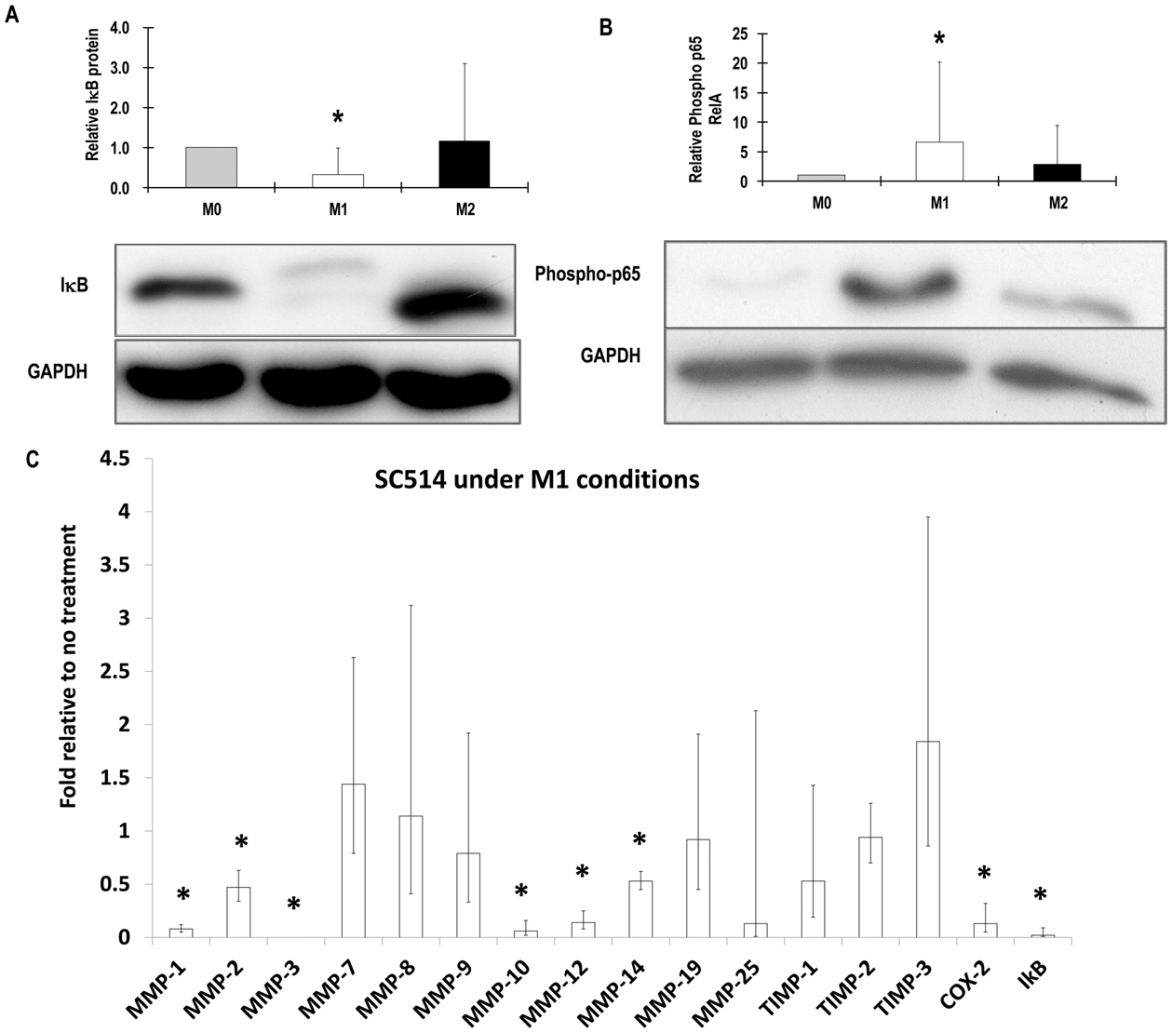
Activation of NF-κB and effects of SC514. M-CSF differentiated macrophages derived from unselected human CD16^+/−^ monocytes (M0) were pre-treated for 45 min with 40 µM SC514 or vehicle (0.1% v/v DMSO) and then some were classically activated with LPS and IFNγ (M1) for 45 minutes. Total IκB proteins and phospho- p65 RelA were measured by western blotting. Levels were normalised to GAPDH as shown. Values are means ± SD * p<0.05 compared to M0 (n = 5). (A) IκB protein (lower band) and phospho- IκB (upper band), (B) Phosho-p65 RelA, (C) Steady-state mRNA levels of MMPs and other genes were measured after 18 hours of stimulation with LPS and IFNγ. Values are log weighted means and 95% confidence intervals * p<0.05 compared to without inhibitor (n = 7).

As expected, M1 treatment stimulated NF-κB activity based on measuring early increases in phospho-IκBα (Fig. 4A) and phospho-p65RelA (Fig. 4B) and later on increased IκBα mRNA levels (Fig. 4C). As expected, IKK2 inhibitor SC514 prevented the rise in IκBα mRNA levels (Fig. 4C). SC514 also decreased expression of the control gene COX-2 and MMPs-1, -2, -3, -10, -12, and -14 (Fig. 4C).

### Role of phosphoinostide-3-kinase (PI-3K)

**Figure 5:**
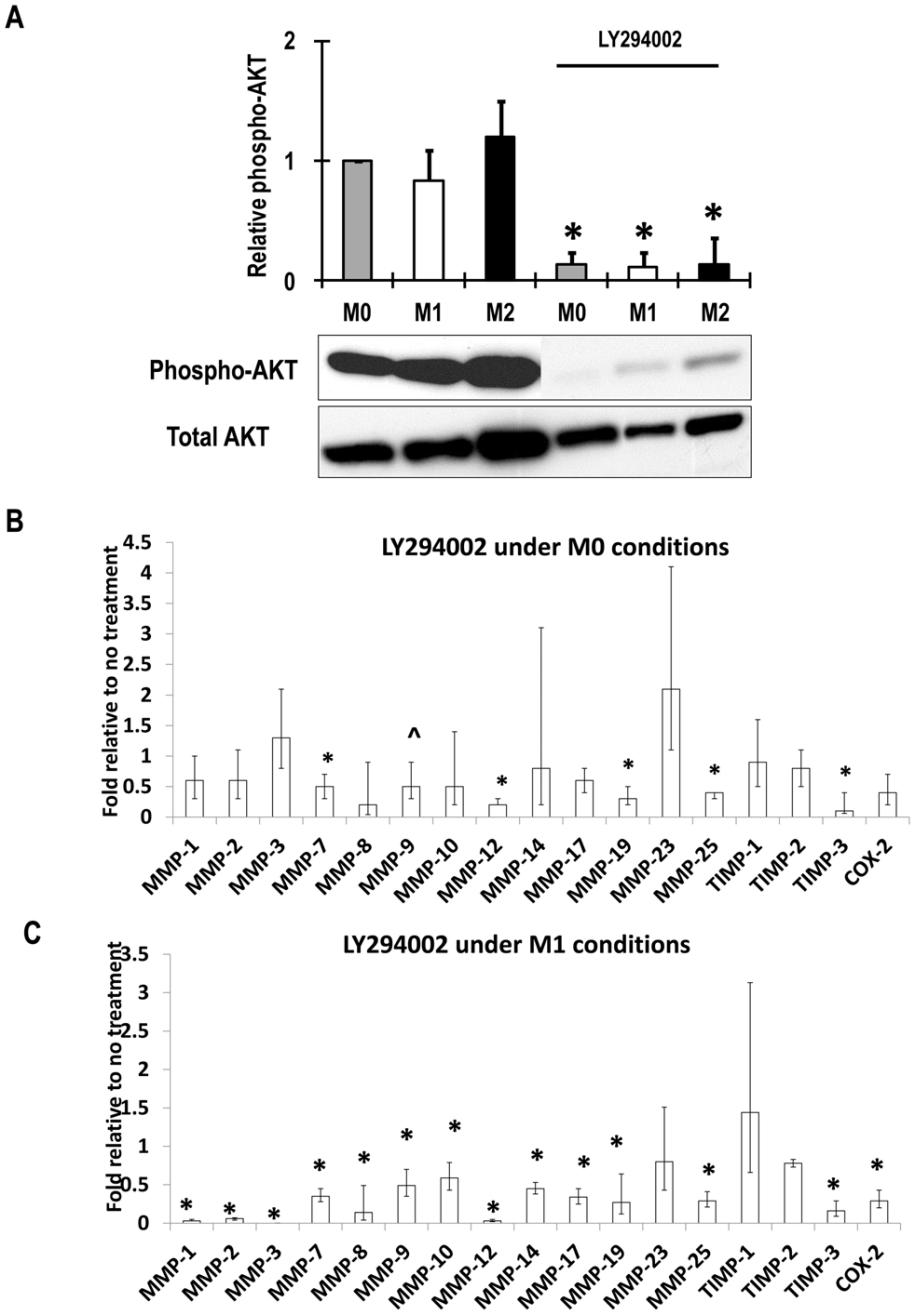
Activation of PI-3K and effects of LY294002. M-CSF differentiated macrophages derived from unselected human CD16^+/−^ monocytes (M0) were pre-treated for 45 min with 10 µM LY294002 and then some were classically activated with for 45 minutes LPS and IFNγ. (A) PI-3-kinase activity as phospho-AKT with and without LY294002 measured by western blotting. Values normalised to total AKT are means ± SD * p<0.05 compared to without inhibitor. (B) Steady-state mRNA levels of MMPs and other genes were measured after 18 hours of no stimulation (M0 conditions). (C) Steady-state mRNA levels of MMPs and other genes were measured after 18 hours of stimulation with LPS and IFNγ (M1 conditions). Panels B and C values are log weighted means and 95% confidence intervals * p<0.05 compared to without inhibitor (n = 7).

Macrophages differentiated in M-CSF had constitutive levels PI-3K activity based on Akt phosphorylation and these were reduced by LY294002 (Fig. 5A). PI-3K inhibitor LY 294002 decreased constitutive mRNA levels of MMP-7, -12, -19 and -25 and TIMP-3 and tended to decrease expression of MMP-9 (p = 0.074), but did not affect the other constitutively expressed MMPs and TIMPs (Fig. 5B). Under M1 conditions LY294002 inhibited all the MMPs, including MMP-9, and TIMP-3 that were inhibited under M0 conditions. LY294002 also inhibited the up-regulation of MMPs-1, -2, -3, -10, and -14 and the M1 marker gene COX-2 (Fig. 5C).

### Expression of MMP proteins in un-stimulated, classically and alternatively activated macrophages

**Figure 6:**
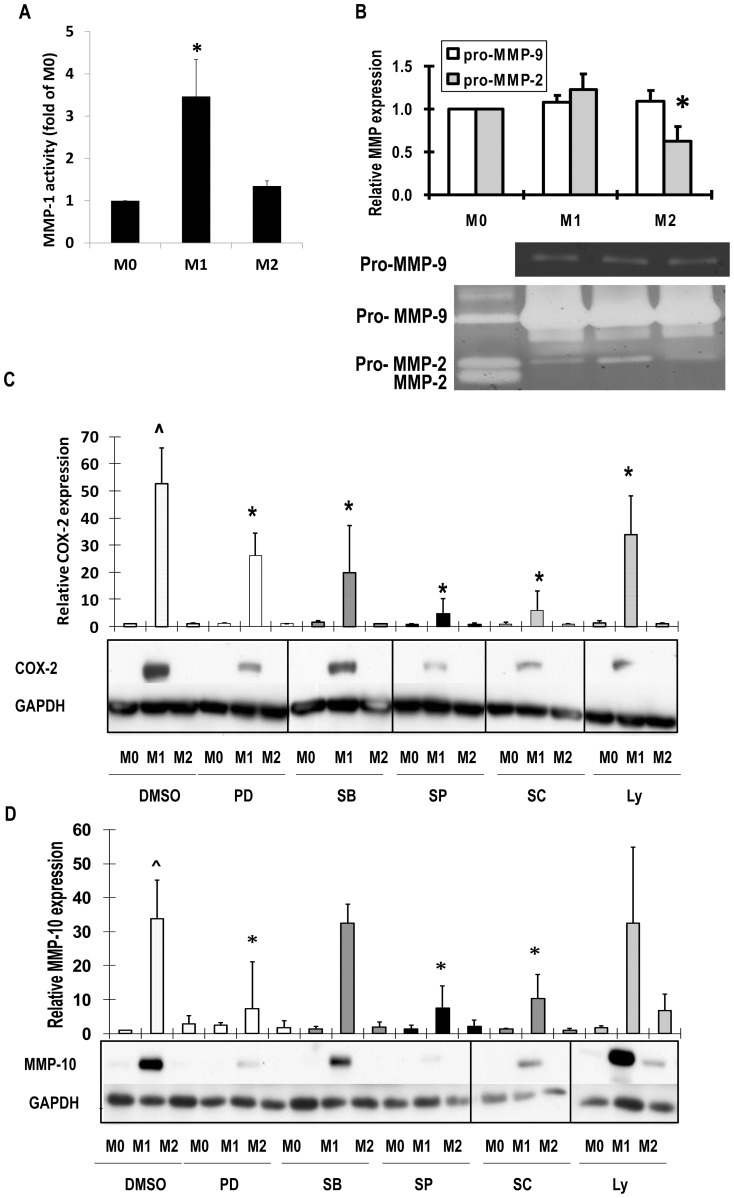
Effects of classical and alternative macrophage activation on MMP protein levels. M-CSF differentiated macrophages derived from unselected human CD16^+/−^ monocytes (M0) or classically activated with LPS and IFNγ (M1) or alternatively activated with IL-4 (M2) for 48 hours. Where indicated cells were pre-treated with 10 µM PD98059 (PD), SB20380 (SB), SP600125 (SP), or LY 294002 (Ly) or 40 µM SC514 (SC) or vehicle (0.1% v/v DMSO). (A) After antibody capture MMP-1 activity was measured with a quenched fluorescent substrate. (B) Zymography for MMP-2 and MMP-9 in conditioned medium. (C) COX-2 in conditioned medium by western blotting. (D) MMP-10 in conditioned medium by western blotting. Values are means ± SD * p<0.05 vs M0 n = 5.

If good assays were available, we confirmed the mRNA expression results at the protein level. For example, using a highly sensitive antibody capture method we showed that MMP-1 activity was relatively low constitutively but increased by classical activation (Fig. 6A). By zymography, gelatinase activity of MMP-2 was much less than MMP-9 and was further suppressed under M2 conditions, consistent with the mRNA data (Fig. 6B). By western blotting, COX-2 and MMP-10 were undetectable in un-stimulated macrophages but were increased in classically activated macrophages (Fig. 6C, D). Moreover, COX-2 protein depended on ERK1/2, p38, JNK, IKK2 and PI-3K (6C), whereas MMP-10 protein depended on ERK1/2, JNK and IKK2 (Fig. 6D), consistent with the mRNA findings (Figs. 3, 4, 5). MMP-12 protein levels were below the limits of detection in un-stimulated and classically-activated macrophages but were stimulated after alternative activation (results not shown), consistent with the greater increase in MRNA levels (Fig. 1B).

### Role of the JAK-2 STAT-1 pathway in MMP and TIMP responses to IFNγ

**Figure 7:**
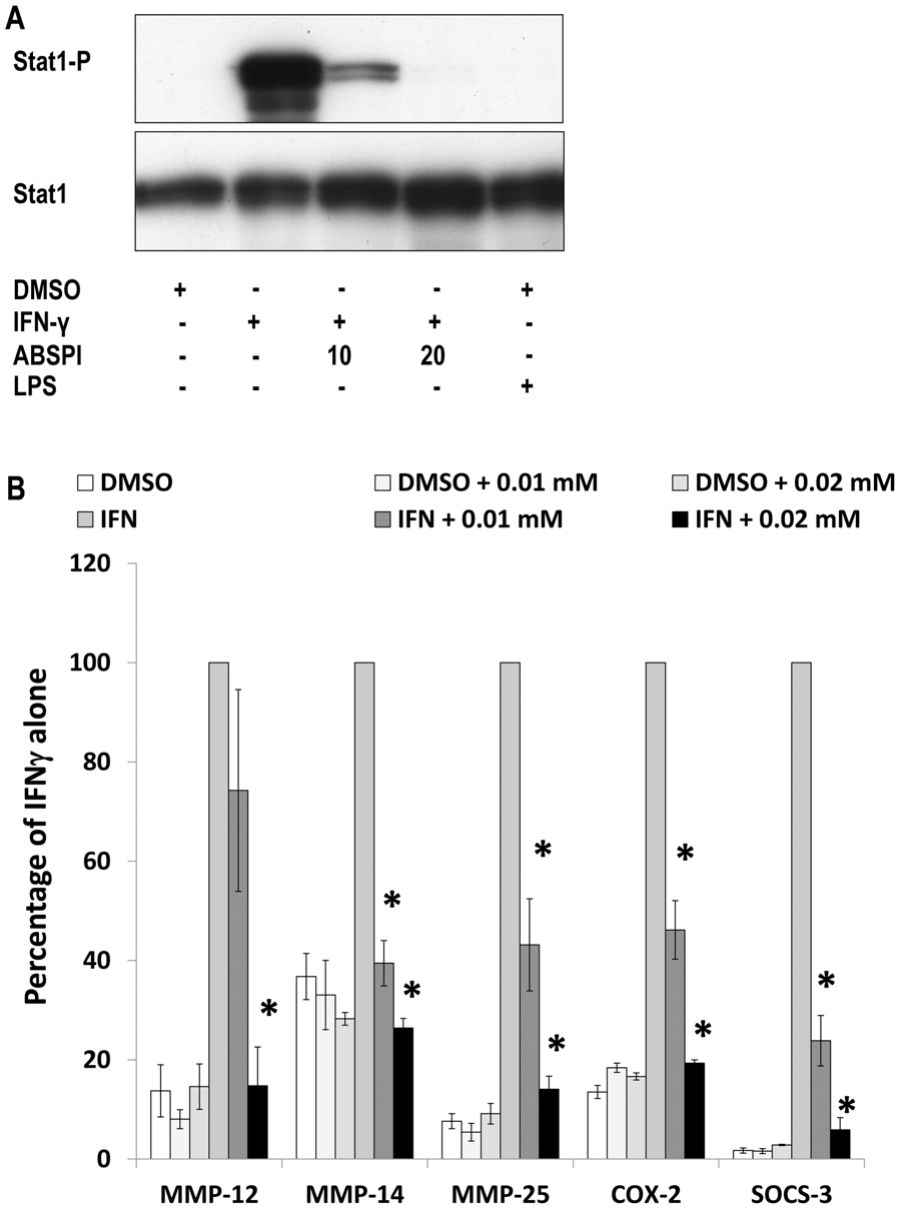
Effects of a JAK-2 inhibitor. M-CSF differentiated macrophages derived from unselected human CD16^+/−^ monocytes were untreated or classically activated with IFNγ. (A) Phospho and total STAT-1 were measured after 45 minutes of IFNγ with and without JAK-2 inhibitor, 10 or 20 µM ABSPI as indicated, or vehicle (0.1% v/v DMSO). (B) Steady-state mRNA levels of MMPs and other genes were measured after 18 hours of stimulation as in panel A. Values are means ± SEM * p<0.05 compared to without inhibitor (n = 4).

Some but not all actions of IFNγ are mediated through janus kinase-2 (JAK-2) [17]. IFNγ activated JAK-2 in macrophages based on STAT-1 phosphorylation and this was inhibited by the low molecular weight inhibitor ABSPI (Fig. 7A). The effects of IFNγ to increase MMP-12, -14 and -25, COX-2 and another positive control gene, SOCS-3, were all inhibited by the JAK-2 inhibitor ABSPI (Fig. 7B). The mRNA levels of MMP-1, -9 and -10 that did not change with IFNγ, were not affected by the JAK-2 inhibitor (not shown), which confirmed the specificity of this treatment.

### Association of MMPs-1 and -10 with classical macrophage activation in atherosclerotic plaques *in vivo*

**Figure 8:**
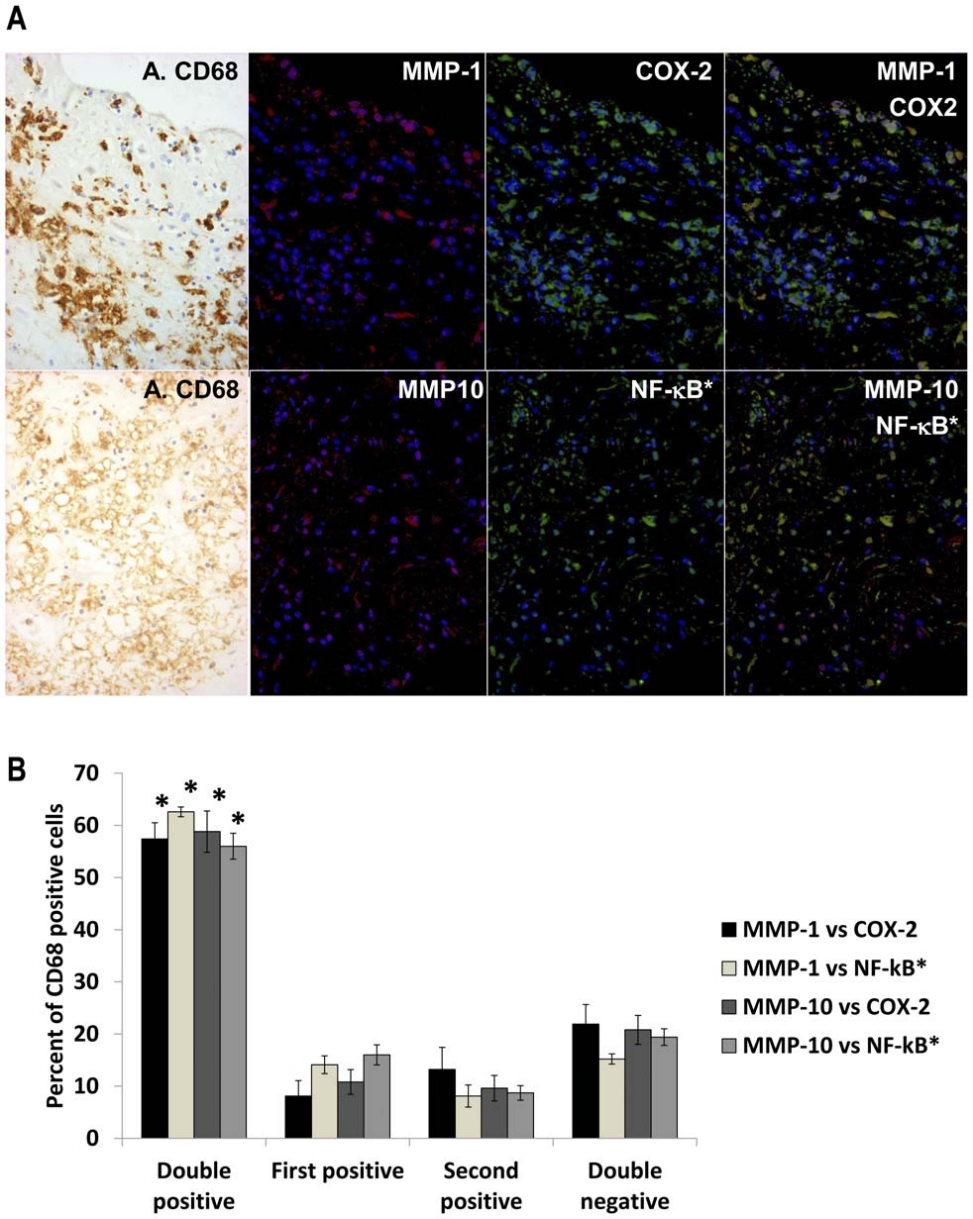
Co-localisation of MMPs with markers of classical activation in human atherosclerotic plaques. (A) Serial sections were stained by peroxidase with anti-CD68 (A. CD68) and by dual immuno-fluorescence with anti-MMP-1 or anti-MMP-10 (red) together with anti-COX-2 or p65RelA (green) as shown, and the images were superimposed digitally. Nuclear localised p65RelA (NF-kB*) was detected because of the shift in nuclear counterstain colour from dark blue (DAPI alone) to sky blue (blue plus green) in the superimposed image. (B) Areas rich in macrophages identified from the peroxidase stain were identified in the serial section. Cells in the whole field were counted and a percentage of each staining pattern calculated. Values are means ± SEM, n = 6, * p<0.05.

Our major *in vitro* findings included that MMP-1 and MMP-10 are up-regulated together with COX-2, the marker of classically activated macrophages, through activation of NF-κB. We took advantage of tissues available from the Bristol Coronary Biobank to validate these findings *in vivo.* Using dual fluorescence immunocytochemistry we showed that over 80% of cells in areas of plaque with abundant CD68 positive macrophages were either double positive for MMP-1 or MMP-10 and COX-2, or else double negative (Fig. 8). Furthermore, over 80% of cells were either double positive for MMP-1 or MMP-10 and nuclear localised NF-κB, or else double negative (Fig. 8).

## Discussion

### Major findings

**Figure 9:**
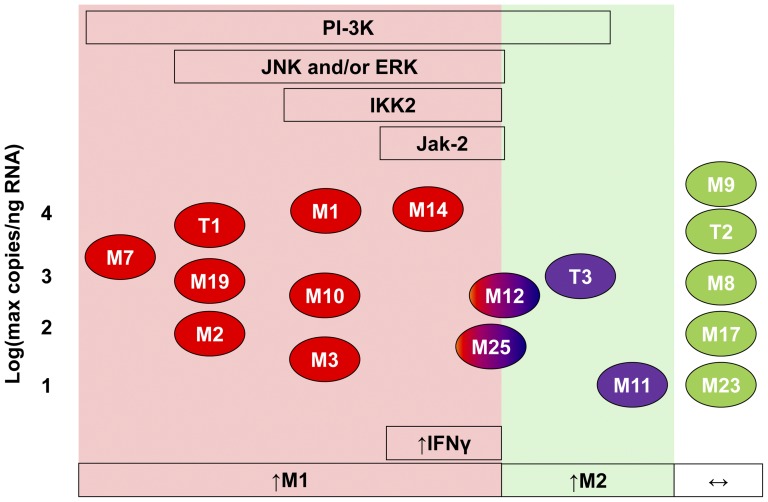
A map of regulation of the MMP system in human macrophages. Pink zone: genes up-regulated by classical activators LPS and IFNγ as shown in the boxes below. Green zone: genes up-regulated during alternative activation (IL-4). White zone: no or minor regulation. Red symbols: up-regulated during classical activation. Blue symbols: up-regulated during alternative activation. Red-blue symbols: similar regulation by classical and alternative activation. Green symbols: scarcely regulated. The boxes above indicate dependence of MMP or TIMP activity on the specified kinases or transcription factors.

Our study is the first to comprehensively survey the MMP system in classically and alternatively activated human macrophages. We demonstrated differential regulation of several MMPs and TIMP-3, including, most excitingly, MMPs -10, -14, -19 and -25 and TIMP-3, which have received little previous attention in the context of inflammation. Moreover we have elucidated the signalling pathways responsible. Fig. 9 sets out a map of the major differences in MMP and TIMP expression and the underlying signalling pathways. In Fig. 9 genes up-regulated during classical activation have red symbols and those down-regulated during alternative activation have blue symbols. A few exceptional genes, MMPs -12 and -25, show up-regulation by both classical and alternative activation and are therefore given red-blue symbols. Genes in the pink zone of the map are up-regulated by classical activators both *in vitro* and in at least one model of human inflammation, the atherosclerotic plaque. Satisfyingly, this group of MMPs depended for their expression on ERK and JNK MAP kinases, PI-3K, and NF-κB, which have been associated previously with classical macrophage activation [5], [18]. The changes mediated selectively by IFNγ rather than other classical activators were all dependent upon JAK-2, which is another new finding. Only MMPs-11 and -12 and TIMP-3 are more up-regulated during alternative activation (Fig. 9 green zone). In the white zone are the MMP and TIMP genes that show no or minor regulation under the conditions of our experiments. Another novel aspect of our study is to consider the impact CD16^−^ and CD16^+^ monocyte populations [16]. We did not find any major consequences of deleting the CD16^+^ population.

### Comparison with previous studies

From previous literature [12], [13] and analysis of expression databases we focussed on those MMPs and TIMPs that have detectable copy numbers of mRNA in human macrophages. In addition we studied MMP-13 and MMP-23 that were identified at high levels in mouse macrophages. Our initial studies used the protocol and resulting marker genes for classical activation with LPS and IFNγ (M1) and for alternative activation with IL-4 (M2) reported in the genomic study of Martinez and colleagues [14]. No MMPs or TIMPs were reported to have a significant, more than 4-fold ratio of expression in M1/M2 conditions in that study [14]. We succeeded, however, because the larger number of replicates we used for our qRT-PCR study mitigated the quite high variability in different blood preparations (Fig. 1, Table 2).

The signalling pathways controlling MMP expression have been previously reviewed [19] and several have been shown to depend on MAP kinases and NF-κB in other cell types [20]. We significantly extended this literature by showing that dependence on ERK1/2 and/or JNK MAP kinases and NF-κB activating kinase, IKK2, is common to all those MMPs and TIMPs that are stimulated by classical macrophage activation, except MMP-7. Interestingly, basal expression of several MMPs (especially MMP-1, -3, and -10) remained very low in the absence of classical activation, despite constitutive ERK1/2 and p38 MAP kinase. This indicates that additional activation of JNK and IKK2 is necessary in concert for MMP up-regulation. Similar conclusions have been reached in several other cell types [20]. The promoters for all the MMPs that are up-regulated by classical activation or decreased by IL-4 during differentiation contain proximal AP-1 or SP-1 transcription factor binding sites [19]. Moreover, the cognate transcription factors depend on MAP kinase activity [21]. Dependency of MMP promoters on NF-κB is less well documented since only MMP-1, -9 and -14 have defined proximal binding sites [19]. The observation in our study that MMP-9 is not induced by classical macrophage activation and that its constitutive production is independent of NF-κB activity is consistent with previous work in human primary macrophages and rabbit foam cell macrophages [22].Nevertheless, well-characterised AP-1 and NF-κB binding sites occur in the MMP-9 promoter and are responsible for synergistic induction of MMP-9 by growth factors and inflammatory cytokines in several other cell types [23], [24]. The basis for the unusual pattern of regulation in macrophages requires further study.

The relative effectiveness of different classical activators on MMP expression levels has not been systematically studied previously. We found here that for most MMPs, the effects of LPS and cytokines were much greater than those of IFNγ. Nevertheless, significant effects of IFNγ were seen on stimulation of MMPs-14 and -25 and inhibition of TIMP-3. MMP-14 and TIMP-3 have been shown to have counter-regulatory roles in macrophage invasion, proliferation and apoptosis [15] and it will be interesting to investigate the role of IFNγ in this switch. Signalling from IFNγ has been shown previously to proceed along JAK-2 dependent and independent pathways [25]. We showed here that all the effects on the MMP system we observed are JAK-2 dependent.

### Functional relevance

There is much literature to support a role for MMPs in various forms of inflammation [2], [3], [8]. For example, Libby's group showed based on histological studies in man and animal experiments, that release of several collagenases form macrophages plays a key role in atherosclerotic plaque rupture (reviewed in [26]). Based on copy numbers of mRNA, we show here that the membrane-type1 MMP, MMP-14, is the most abundantly-expressed collagenase of un-stimulated human macrophages (Fig. 1, 9). Our data from mRNA and protein measurements demonstrated that MMP-14 is further increased by classical activation, which appears to be a novel finding (see review in [4]). MMP-14 has been shown to mediate ICAM-dependent migration of monocytes across endothelial cell layers [27] and to promote the invasion of macrophages and foam cell macrophages through matrigel [15]. The secreted collagenase MMP-1 is also greatly up-regulated upon classical activation and this led to an increase in collagenase activity (Fig. 6A). Furthermore we showed that MMP-1 co-localised with COX-2, the marker of classical macrophage activation, in human atherosclerotic plaques. By contrast, the so-called neutrophil collagenase, MMP-8, was down-regulated under M1 conditions in our study. Although abundant in mouse macrophages (reviewed in [4]), we could not detect MMP-13 expression in human macrophages when using 3 different sets of validated PCR primers, in agreement with a previous study [12]. MMP-19 is also considered a collagenase, although from mouse knockout studies [28], tenascin-C appears to be an important substrate *in vivo*. MMP-19 has been previously identified as a cell-surface associated MMP in monocytes stimulated by adhesion [29] or chemokine CCR5 but not LPS [30]. We now add that it is abundant in un-stimulated macrophages. Knockout of MMP-19 in mice leads to complex phenotypes owing to a defect in thymocyte maturation [31]. Nevertheless, future studies to identify its function in inflammatory conditions including atherosclerosis appear warranted.

The gelatinases, MMP-2 and MMP-9 have unique gelatin binding domains inserted into their catalytic domains [1]. We show here that MMP-9 is much more abundant than MMP-2 in differentiated human macrophages based on mRNA levels and zymography.

Among the stromelysins, MMP-3 has been quite widely studied in the context of inflammation [2] but role of MMP-10 is much less appreciated. MMP-10 is stimulated in human brain microglia by amyloid beta peptide [32] and recent transcriptomic studies identified MMP-10 as up-regulated among other genes during inflammation in human chondrocytes and adipocytes [33], [34]. MMP-10 can activate the pro-form of MMP-1 [35] and hence the similar substantial up-regulation of MMP-1 and -10 *in vitro* and their co-localisation in atherosclerotic plaques (Fig. 8) could be significant for collagen degradation. Indeed cleavage of collagen has long been known to co-localise with MMP-1 expression in human atherosclerotic plaques [36].

The matrilysin MMP-7, which lacks a C terminal haemopexin domain, was abundant in un-stimulated macrophages and further up-regulated during classical activation, consistent with its previously identified roles in inflammation [2].

Interestingly, we found that the much less studied membrane-type metalloproteinase MMP-25 (MT6-MMP) is induced by classical and alternative activation, although the effects of classical activation are stronger. MMP-25 was originally cloned and named leukolysin because of its restriction to blood leukocytes and bone marrow [37]. The alpha-1-proteinase inhibitor [38] and myelin basic protein [39] have been identified as efficient substrates of MMP-25 and a role for the enzyme has been proposed in autoimmune multiple sclerosis [39]. MMP-25 was shown to be up-regulated by LPS in mouse macrophages [39] but regulation in humans has not apparently been reported. Our results prompt more research into this MMP.

In great contrast to the other MMPs, the matrix metalloelastase, MMP-12, appears to be more readily up-regulated in alternatively than classically-activated macrophages. A prominent role for MMP-12 in alternative activated macrophages has been implied previously during aneurysm formation in mice [40].

TIMP-1, -2 and -3 were expressed at relatively high copy numbers in un-stimulated macrophages *in vitro* and TIMP-3 was down-regulated by classical activation, which implies a further increase in the ratio of MMP gene expression to that of TIMPs. Conversely, TIMP-3 was increased by alternative activation, which appears to be a novel finding (see review in [4]).

### Conclusion

In summary, Fig. 9 maps out the divergent regulation of the MMPs and TIMPs during classical and alternative activation of human macrophages. Furthermore, it demonstrates how differential dependence on ERK1/2 and JNK MAP kinases, NF-κB activating kinase IKK2, PI-3K and JAK-2 activation underlie this regulation. Our mechanistic studies should help future work aimed at modulating MMP expression and thereby preserving the ECM in diseases such as rheumatoid arthritis, periodontal disease atherosclerosis and aneurysms, which result from excessive classical or alternative macrophage activation.
